# Gender Differences and Performance Changes in Sprinting and Long Jump Among Young Athletes

**DOI:** 10.3390/sports12120347

**Published:** 2024-12-16

**Authors:** Eduard Bezuglov, Evgeniy Achkasov, Timur Vakhidov, Georgiy Malyakin, Elizaveta Kapralova, Vyacheslav Kolesnichenko, Mikhail Vinogradov, Tatiana Zharikova, Anton Emanov

**Affiliations:** 1 High Performance Sports Laboratory, Sechenov First Moscow State Medical University, 119991 Moscow, Russia; e.n.bezuglov@gmail.com (E.B.); vakhidovt@yandex.ru (T.V.); malyakin_g_i@staff.sechenov.ru (G.M.); hitrec.ru@mail.ru (V.K.); vinogradov.coach@gmail.com (M.V.); emanov_a_yu@staff.sechenov.ru (A.E.); 2 Department of Sports Medicine and Medical Rehabilitation, Sechenov First Moscow State Medical University, 119991 Moscow, Russia; achkasov_e_e@staff.sechenov.ru; 3 Department of Human Anatomy and Histology, Sechenov First Moscow State Medical University, 119991 Moscow, Russia; zharikova_t_s@staff.sechenov.ru

**Keywords:** gender divergence, sprint, athletics, performance, growth spurt

## Abstract

This research aimed to examine the dynamics of changes in sprint and long jump performance and the age of gender divergence in well-trained young athletes specialising in athletics. Data were collected from 1249 male and 1497 female athletes aged 10 to 15 years who participated in the final round of the annual national athletics tournament “Shipovka Yunykh” from 2017 to 2019. The top 50 results in each age group for the 60-metre sprint and long jump were analysed. Boys showed significantly higher performance than girls in both events from ages 11 and 12. Significant performance improvements were noted between ages 12 and 13 for boys in the long jump and 10–11 in the sprint. Girls showed significant improvements between ages 12 and 13 in the long jump and 11–12 in the sprint. Gender divergence in sprint and long jump performance occurs around age 11. The dynamics of performance changes are influenced by the timing of the growth spurt, highlighting the need for age-specific competition categories in athletics.

## 1. Introduction

In recent decades, the popularity of several sports, including soccer, ice hockey, and athletics, has significantly increased [1,2]. This has led to more children participating in these sports, increased levels of competition, and higher training costs for potentially successful adult athletes. As a result, many leading sports organisations are implementing talent identification programmes. These programmes aim to individualise the career trajectory of each potentially successful adult-level athlete and minimise the discriminatory impact of various sociocultural and biological factors. These factors include the relative age effect, self-fulfilling prophecies (Galatea, Pygmalion, and Golem effects), and the different chronological ages of the growth spurt onset [3,4,5].

Unlike soccer and ice hockey, athletics typically has a relatively late start of regular organised training (usually no earlier than 9–10 years). There are also fewer competitions in childhood and adolescence, with age groups including athletes born in at least two consecutive calendar years. Another important feature of early-stage athletics competitions is the predominance of disciplines where key success factors are strength and speed, with technique playing a relatively minor role due to limited training experience [6,7,8].

For older athletes with an earlier growth spurt and, therefore, an earlier increase in serum testosterone concentration, an increase in growth and limb length may contribute to enhanced performance. According to high-quality scientific data, the growth spurt in boys begins at ages 12–13 and lasts about two years [9,10,11]. This is most likely related to the now proven influence of endogenous testosterone on strength, speed, endurance, and bone density [12,13,14,15]. Testosterone can be called the strongest factor explaining male advantage in sports results, most often ranging from 10 to 20% [16,17,18]. In sports where muscle mass and explosive strength are key to success, this difference can reach 50% [19]. Additionally, a more advanced biological age may mean not only that they are more mature, but also that they have more experience interacting with the environment and more training experience [3,20]. Both of these factors can influence the success of athletes in their age groups. Although this period can be accurately determined using both non-invasive and invasive methods (radiography or ultrasound of the left hand bones), these methods are rarely used in sports organisations’ practices [21,22,23].

Currently, there are only two studies examining the dynamics of changes in sports performance and the age of gender divergence in well-trained young athletes of both sexes in athletics. One study among Norwegian athletes aged 11–18 concluded that young male and female athletes perform almost equally in running and jumping events until age 12 [24]. Importantly, the authors of this study noted that it was “the first study to present absolute and relative annual performance developments in running and jumping events for competitive athletes from early to late adolescence”.

In another study published in 2024, the authors analysed the top 50 elite American boys and girls aged 7–18 in athletics events (100 m, 200 m, 400 m, 800 m, long jump, and high jump) and found a significant performance difference (about 5%) between sexes in all disciplines in each age group starting from age seven [25].

Thus, existing results are few and contradictory, necessitating studies on the dynamics of sprint and long jump performance changes in well-trained young athletes specialising in athletics of both sexes before, during, and after the growth spurt. The hypothesis of this study suggests that performance improvements between certain age groups will vary by gender, reflecting the timing of growth spurts. This will allow for a more objective assessment of an athlete’s potential at specific times and the correct dosage of training load for young athletes of different ages.

## 2. Materials and Methods

This study employed a retrospective design. Data were collected on the sports performance of young athletes specialising in athletics. The information was gathered during the final round of the prestigious annual national athletics tournament ‘Shipovka Yunykh’ from 2017 to 2019. During these competitions, winners are determined by the sum of results in three sports disciplines, including the 60-metre sprint and long jump. During the specified period, the competitions were held at stadiums meeting international standards, under similar weather conditions each year. Results in running events were determined using an electronic timing system, and judging was conducted by certified referees.

The sample consisted of the results of 1249 male and 1497 female athletes. The distribution of male and female athletes by the analysed disciplines was as follows: 701 and 859 in the 60-metre sprint, and 548 and 620 in the long jump, respectively. The top 50 results in each age group of young athletes aged 10–15 who participated in the final round were used for analysis. The top 50 results in each age group were selected to analyze elite-level performance while ensuring a representative sample size for statistical analysis. This approach focuses on well-trained young athletes consistently competing at the highest level, enabling targeted evaluation of performance trends, including gender divergence and growth-related changes in sprint and long jump.

The study was conducted in accordance with the Helsinki Declaration and was approved by the Local Ethics Committee of the University [protocol № was removed for the blind review]. All data used are publicly available, so participant consent was not required.

### Statistical Analysis

Data analysis was conducted using jamovi, version 1.8.2. The Kolmogorov–Smirnov test determined variable distribution normality. The mean value, standard deviation, and 95% confidence interval were reported for normally distributed variables. ANOVA followed by Tukey’s post-hoc test compared results among boys and girls of the same age, girls of different ages, and boys of different ages. Results were considered significant at *p* < 0.05.

## 3. Results

The chronological age of athletes in most age groups did not show significant differences. Only in the 13-year-old sprint group and the 15-year-old jump group were boys significantly older than girls (*p* = 0.012 and *p* = 0.031, respectively) (Table 1).

In the sprint, boys had significantly higher results than girls starting from age 12. Before this age, the results did not differ significantly. In the long jump, boys showed significantly higher results in all age groups starting from age 11 (Table 2).

When comparing the sprint results of girls in all age groups, significant differences were found up to the transition from 14 to 15 years, with older girls performing better. The exception was the 14 and 15-year-old age groups, where no significant difference was found. The greatest improvement in results was noted between the 10 and 11-year-old age groups (Table 3).

Identical dynamics of age-related changes in results were observed when comparing the long jump results of girls in different age groups. However, the greatest improvement in this discipline was between ages 12 and 13 (Table 4).

Among boys, significant differences in sprint results were observed across all age groups, with results improving at older ages. The greatest improvement in results was found between ages 12 and 13 (Table 5).

In the analysis of long jump results among boys, significant differences were found in all age groups, with better results at older ages. The greatest improvement in results was between ages 13 and 14 (Table 6).

The table of normative values was developed based on a statistical analysis of the top 10 best performances. These values serve as a benchmark for comparing athletes’ results with those of the leading performers in their sport (Table 7).

## 4. Discussion

The analysis of the obtained data allows the primary conclusion that as chronological age increases, sprint and long jump results (the disciplines most significantly associated with strength and serum testosterone concentration) improve. Furthermore, the performance difference between athletes born in two consecutive years is statistically significant. Therefore, separating groups consisting of athletes born in different years significantly reduces the likelihood of athletic success for chronologically younger athletes specialising in athletics.

The decision to focus on the 60-metre sprint and long jump disciplines is driven by both practical and theoretical considerations. Practically, the availability of events in the competition “Shipovka Yunykh” is limited to four core athletic disciplines—sprint, long jump, middle distance, and throwing. The sprint and long jump were selected as they represent speed- and strength-dominated events, which are highly sensitive to the growth spurt and testosterone levels during adolescence.

Theoretically, sprint and long jump performance are closely tied to key physical attributes that develop significantly during puberty. Both disciplines rely on explosive strength, speed, and neuromuscular coordination, all of which are influenced by increases in testosterone and rapid skeletal growth during the adolescent growth spurt (Largo et al., 1978; Tanner 1986). This makes them ideal for investigating gender differences in performance, as the growth spurts in boys and girls occur at different times and at different rates, leading to distinct performance trajectories during this period.

Furthermore, previous studies have demonstrated that these disciplines are among the first to show significant gender divergence in performance due to physiological differences in muscle mass and hormonal changes [24,26]. The reliance of these disciplines on strength and speed, both of which are significantly impacted by biological maturation, makes them ideal for the objectives of this study. In the sprint, the most vulnerable groups are boys aged 12 and girls aged 10, as the most pronounced improvement in results for boys occurs between 12 and 13 years (−0.472 s), and for girls between 10 and 11 years (−0.287 s). In the long jump, the most disadvantaged are girls aged 12, as the most significant improvement in results (+28.5 cm) occurs between 12 and 13 years. This is likely related to the growth spurt described in several previous studies [9,10,11].

The results also showed that boys have significantly higher sprint results starting at age 12. In the long jump, gender divergence in results appears as early as age 11.

To our knowledge, this is one of the few studies examining the absolute and relative changes in long jump and sprint results of the best young competitors in athletics during adolescence. The first such study was published by Tønnessen et al. [15], and two more by Handelsman et al. and Atkinson et al. in 2017 and 2024, respectively [16,18]. Another study by Malina et al. [27] included over 300 students from a city athletics school aged 11–15 years. The authors of this study found gender differences in sports performance in all age groups starting at age 11, consistent with the data obtained by Atkinson et al. [25].

Our findings align with previous research that emphasises the importance of technical factors such as take-off mechanics in predicting athletic success. For example, Ozaki and Ueda found that minimising deceleration at take-off and optimising relative vertical momentum are key to high-level performance in jumping events [28]. These technical aspects are influenced by the timing of biological growth spurts, which also explains the performance improvements observed between ages 12 and 13 in our study.

Similarly, Rodriguez-Gomez et al. demonstrated in a long-term analysis of elite Spanish jumpers [29] that performance success is closely linked to the development of specific physical and technical attributes during adolescence. Our study extends this understanding by showing how these performance trends vary between genders during key stages of maturation.

As mentioned above, the existing results can be considered contradictory, but in two studies involving elite young athletes specialising in athletics, strict evidence was obtained that there is an age before which boys’ and girls’ results in the analysed disciplines are comparable [24,30].

The data obtained in this study also support Tønnessen et al.‘s [24] conclusions regarding the age of gender divergence in results, but the most significant improvement in sprint and jump results for girls differed. Among the participants of “Shipovka Yunykh”, it was between ages 10 and 11 for the sprint and between ages 12 and 13 for the long jump. The age of the most significant improvement in boys’ sprint and long jump results was similar to the data of Norwegian specialists: between ages 12 and 13 and 13 and 14, respectively. The data obtained allow a confident conclusion that up to a certain age, boys and girls participating in high-level competitions demonstrate comparable results, at least in the 60-metre sprint and long jump.

Thus, the results of the conducted study confirm the hypothesis that the dynamics of changes in sprint and long jump performance among young male and female athletes aged 10 to 15 vary depending on the age group. This may reflect the influence of the timing of the growth spurt on these changes.

Before the puberty period, speed–strength indicators do not differ between boys and girls, as their testosterone levels are equally low and range from 0.2 to 0.7 nmol/L in children under 10 years of age. From the beginning of the puberty period (growth spurt), testosterone levels in boys sharply increase and can rise by 30 times compared with pre-puberty levels, and in adult men, it is approximately 15 times higher than in women of the same age, reaching 40 nmol/L. In girls, testosterone concentration also increases but to a much lesser extent. According to Senefeld et al. [31], the average testosterone concentration in American girls aged 6 to 20 increases from 0.08 nmol/L to 1.02 nmol/L, reaching a plateau starting at age 14, while their male peers’ concentration increases from 0.07 nmol/L to 17.9 nmol/L at age 20, reaching a plateau starting at age 17. An even more significant difference was found in the study by Bezuglov et al. [26] involving elite young competitors in athletics aged 17–18. Boys’ serum testosterone concentration was 26.4 ± 9.6 nmol/L, while girls’ was 1.5 ± 0.7 nmol/L.

After age 12–13, testosterone levels in adolescents begin to increase with an uneven rate that can vary significantly among adolescents of the same chronological age. In this situation, young athletes with higher biological maturity and, therefore, higher testosterone levels at a specific time have an advantage. At ages 16–18, testosterone concentration reaches a plateau, and the advantage determined by testosterone ceases to play a key role.

However, by this time, many “late-maturing” athletes specialising in athletics have already stopped competing or are not considered by coaches as potentially successful adult-level athletes [32]. However, there are at least two other factors that can influence the athletic success of chronologically older track and field athletes. These include a longer period of regular training and the Matthew effect, which can occur when older and, at some point, more successful young athletes are placed in better sporting organisations and trained by better coaches using best practices [3,33,34].

This study’s results also provided normative values in the 60-metre sprint and long jump for elite young competitors in athletics. To our knowledge, such data for young athletes of a comparable level specialising in athletics have not been published in the accessible scientific literature until now. Thus, for elite young track and field athletes, normative values for a particular distance and discipline are provided only in the studies of Atkinson et al., 2024, in which disciplines such as 100 m, 200 m, 400 m, 800 m, long jump, and high jump were studied. Normative values are also provided for 20 m and 30 m in two other studies [27,35]. This information can help practitioners correctly interpret athletes’ current results and assess their progress.

The study also demonstrated that the results of athletes in adjacent age groups differ significantly, and in athletics, young athletes compete in age groups that include at least two consecutive years of birth. This may lead to reduced potential athletic success for chronologically younger athletes (especially late-maturing ones) in the youngest age groups of 10–15 years, as this period corresponds to the growth spurt associated with a sharp increase in serum testosterone concentration in boys and a significantly less pronounced but still statistically significant increase in girls [26,30,36,37]. Many scientific studies show that later-born young athletes (chronologically youngest in competitive age groups) have a much higher chance of success at the adult elite level, including in soccer, ice hockey, and athletics [38,39,40].

While current athletics competitions are structured by calendar age groups, the findings of this study provide valuable insights for coaches and talent scouts in developing more individualised approaches to athlete development. The performance improvements seen between certain ages—particularly the divergence of boys’ and girls’ results around age 11—highlight the critical role of biological growth in shaping athletic potential. These insights suggest several practical applications:

1. Individualised training programmes: Coaches should recognise that performance improvements during adolescence may be influenced more by biological maturity than by chronological age. Athletes who mature early or late may require individualised training plans that account for their unique growth trajectory. This could involve adjusting training loads, particularly in strength- and speed-focused disciplines such as sprinting and long jump, to align with the athlete’s stage of physical development.
2. Talent identification and development: Talent identification programmes can use these findings to better evaluate athletes’ long-term potential. Rather than focusing solely on current performance, it is important to consider an athlete’s growth phase and potential for future development. Late-maturing athletes may not exhibit peak performance in early adolescence but could excel later if provided with appropriate support and training opportunities. This requires a shift in scouting strategies to look beyond immediate results and assess an athlete’s developmental trajectory.
3. Mitigating disadvantages for younger athletes: As this study shows that younger athletes in a given age group (those closer to the beginning of the calendar year) may be at a disadvantage, coaches should be cautious not to overlook late developers. Individualised assessment methods could be introduced in training environments to ensure that younger or late-maturing athletes are not discouraged or overlooked in talent development pipelines.

Overall, this study provides evidence that early adolescence is a critical period for physical development in athletics, and by acknowledging the impact of biological maturation, practitioners can better guide young athletes toward long-term success in the sport.

Future studies should consider the influence of maturity status on athletic success, and normative values of various indicators should be stratified not only by age but also by the degree of somatic maturity.

### Limitations

The limitations of this study include its retrospective design, which did not account for the possible influence of maturity status on the results. It is very likely that young athletes specialising in athletics achieving success in early adolescence are most often early-maturing, and their results cannot be extrapolated to other sports.

A significant limitation of the present study is the absence of direct measures of biological age or skeletal maturity, such as sitting height, standing height, weight, or skeletal age, which are important for understanding growth and maturation processes in adolescent athletes. While chronological age was used to categorise participants, it is well established that biological age plays a crucial role in athletic development and performance, particularly during adolescence when hormonal changes significantly impact physical capabilities [32].

Due to the publicly available nature of the dataset and logistical constraints, collecting detailed biological age indicators for the athletes in this study was not feasible. Future studies should aim to include biological markers such as skeletal age or sitting height to better understand how maturation affects performance across different age groups. This could provide a more nuanced understanding of how biological maturity impacts the dynamics of sprint and long jump performance.

## 5. Conclusions

Gender divergence in sprint performance among well-trained young male and female athletics competitors occurs no earlier than age 11, and the dynamics of their changes differ significantly and are likely dependent on the timing of the growth spurt. The data convincingly demonstrate that to maintain fair competition, young athletics competitors should compete in age groups that include one calendar year, similar to the most competitive early specialisation sports such as soccer and ice hockey.

## Figures and Tables

**Table 1:** Mean chronological age (in months) by age group in analysed disciplines.

Age Group	Sprint			Jump		
	Boys	Girls	*p*-Value	Boys	Girls	*p*-Value
10 years	127 ± 3.1, n = 92	127 ± 3.1, n = 96	0.78	127 ± 3.2, n = 111	127 ± 3.2, n = 104	0.46
11 years	138 ± 3.0, n = 104	138 ± 3.0, n = 102	0.69	138 ± 3.2, n = 90	137 ± 3.3, n = 125	0.93
12 years	151 ± 3.4, n = 94	151 ± 3.15, n = 144	0.36	152 ± 3.1, n = 92	151 ± 3.1, n = 111	0.12
13 years	163 ± 3.0, n = 108	162 ± 3.4, n = 216	0.012	162 ± 3.1, n = 98	162 ± 3.3, n = 117	0.19
14 years	175 ± 3.2, n = 148	174 ± 3.3, n = 185	0.063	175 ± 3.1, n = 78	174 ± 3.5 n = 95	0.13
15 years	184 ± 2.6, n = 155	184 ± 2.7, n = 116	0.30	184 ± 2.6, n = 79	183 ± 2.4, n = 86	0.031
10 years	127 ± 3.1, n = 92	127 ± 3.1, n = 96	0.78	127 ± 3.2, n = 111	127 ± 3.2, n = 104	0.46

**Table 2:** Mean results (top 50) of athletes of both sexes by age group in analysed disciplines. Results in seconds for the sprint and in centimetres for the jump.

Age Group	Sprint			Jump		
	Boys	Girls	*p*-Value	Boys	Girls	*p*-Value
10 years	9.19 ± 0.20, n = 92	9.17 ± 0.19, n = 96	0.48	403 ± 22, n = 111	403 ± 19, n = 104	0.98
11 years	8.93 ± 0.21, n = 104	8,93 ± 0.19, n = 102	0.83	434 ± 22, n = 90	423 ± 19, n = 125	<0.001
12 years	8.48 ± 0.19, n = 94	8.64 ± 0.15, n = 144	<0.001	460 ± 22, n = 92	448 ± 18.5, n = 111	<0.001
13 years	8.01 ± 0.15, n = 108	8.52 ± 0.14, n = 216	<0.001	514 ± 24.5, n = 98	476 ± 18, n = 117	<0.001
14 years	7.76 ± 0.15, n = 148	8.40 ± 0.14, n = 185	<0.001	570 ± 30, n = 78	493 ± 17.5 n = 95	<0.001
15 years	7.64 ± 0.14, n = 155	8.33 ± 0.15, n = 116	<0.001	594 ± 30, n = 79	489 ± 22, n = 86	<0.001

**Table 3:** Average difference in results (top 50) among girls of different age groups in sprint discipline. Results in seconds for the sprint.

	11 Years	12 Years	13 Years	14 Years	15 Years
10 years	0.287 *p* < 0.001	0.528 *p* < 0.001	0.649 *p* < 0.001	0.776 *p* < 0.001	0.8384 *p* < 0.001
11 years		0.241 *p* < 0.001	0.362 *p* = 0.816	0.489 *p* < 0.001	0.5512 *p* < 0.001
12 years			0.121 *p* < 0.001	0.248 *p* < 0.001	0.3106 *p* < 0.001
13 years				0.127 *p* < 0.001	0.1895 *p* < 0.001
14 years					0.0627 *p* = 0.055

**Table 4:** Average difference in results (top 50) among girls of different age groups in long jump discipline. Results in centimetres for the jump.

	11 Years	12 Years	13 Years	14 Years	15 Years
10 years	−19.5 *p* < 0.001	−44.6 *p* < 0.001	−73.1 *p* < 0.001	−91.8 *p* < 0.001	−89.73 *p* < 0.001
11 years		−25.1 *p* < 0.001	−53.6 *p* < 0.001	−72.3 *p* < 0.001	−70.23 *p* < 0.001
12 years			−28.5 *p* < 0.001	−47.2 *p* < 0.001	−45.09 *p* < 0.001
13 years				−18.7 *p* < 0.001	−16.61 *p* < 0.001
14 years					2.09 *p* = 0.98

**Table 5:** Average difference in results (top 50) among boys of different age groups in sprint discipline. Results in seconds for the sprint.

	11 Years	12 Years	13 Years	14 Years	15 Years
10 years	0.302 *p* < 0.001	0.712 *p* < 0.001	1.185 *p* < 0.001	1.435 *p* < 0.001	1.547 *p* < 0.001
11 years		0.411 *p* < 0.001	0.883 *p* < 0.001	1.133 *p* < 0.001	1.246 *p* < 0.001
12 years			0.472 *p* < 0.001	0.722 *p* < 0.001	0.835 *p* < 0.001
13 years				0.250 *p* < 0.001	0.363 *p* < 0.001
14 years					0.113 *p* < 0.001

**Table 6:** Average difference in results (top 50) among boys of different age groups in long jump discipline. Results in centimeters for the jump.

	11 Years	12 Years	13 Years	14 Years	15 Years
10 years	−35.7 *p* < 0.001	−56.4 *p* < 0.001	−110.5 *p* < 0.001	−167 *p* < 0.001	−191.0 *p* < 0.001
11 years		−20.8 *p* < 0.001	−74.9 *p* < 0.001	−132 *p* < 0.001	−155.4 *p* < 0.001
12 years			−54.1 *p* < 0.001	−111 *p* < 0.001	−134.6 *p* < 0.001
13 years				−56.7 *p* < 0.001	−80.5 *p* < 0.001
14 years					−23.8 *p* < 0.001

**Table 7:** Performance benchmarks of top 10 athletes (normative values).

Age Group	Boys Sprint	Girls Sprint	*p*-Value Sprint	Boys Jump	Girls Jump	*p*-Value Jump
10 years	8.77 ± 0.11, n = 10	8.79 ± 0.16, n = 10	0.761	379.00 ± 1.15, n = 10	379.80 ± 0.63, n = 10	0.075
11 years	8.43 ± 0.07, n = 10	8.47 ± 0.11, n = 10	0.346	414.20 ± 1.55, n = 10	403.00 ± 0.94, n = 10	0.000
12 years	8.11 ± 0.10, n = 10	8.28 ± 0.11, n = 10	0.002	432.20 ± 1.32, n = 10	428.10 ± 0.88, n = 10	0.000
13 years	7.70 ± 0.07, n = 10	8.16 ± 0.10, n = 10	0.000	488.90 ± 0.88, n = 10	456.10 ± 0.88, n = 10	0.000
14 years	7.44 ± 0.09, n = 10	8.09 ± 0.08, n = 10	0.000	541.00 ± 1.41, n = 10	473.20 ± 1.55, n = 10	0.000
15 years	7.34 ± 0.08, n = 10	8.01 ± 0.05, n = 10	0.000	561.30 ± 1.89, n = 10	467.30 ± 1.06, n = 10	0.000

## Data Availability

The datasets used and/or analysed during the current study are available from the corresponding author on reasonable request.
